# Improving educational achievement and anaemia of school children: design of a cluster randomised trial of school-based malaria prevention and enhanced literacy instruction in Kenya

**DOI:** 10.1186/1745-6215-11-93

**Published:** 2010-10-07

**Authors:** Simon Brooker, George Okello, Kiambo Njagi, Margaret M Dubeck, Katherine E Halliday, Hellen Inyega, Matthew CH Jukes

**Affiliations:** 1 Malaria Public Health & Epidemiology Group, Kenya Medical Research Institute-Wellcome Trust Research Programme, Nairobi, Kenya; 2 Faculty of Infectious and Tropical Diseases, London School of Hygiene and Tropical Medicine, London, UK; 3 Division of Malaria Control, Ministry of Public Health and Sanitation, Nairobi, Kenya; 4 School of Education, Health and Human Performance, College of Charleston, Charleston, USA; 5 College of Education and External Studies, University of Nairobi, Nairobi, Kenya; 6 Graduate School of Education, Harvard University, Cambridge, Massachusetts, USA

## Abstract

**Background:**

Improving the health of school-aged children can yield substantial benefits for cognitive development and educational achievement. However, there is limited experimental evidence on the benefits of school-based malaria prevention or how health interventions interact with other efforts to improve education quality. This study aims to evaluate the impact of school-based malaria prevention and enhanced literacy instruction on the health and educational achievement of school children in Kenya.

**Design:**

A factorial, cluster randomised trial is being implemented in 101 government primary schools on the coast of Kenya. The interventions are (i) intermittent screening and treatment of malaria in schools by public health workers and (ii) training workshops and support for teachers to promote explicit and systematic literacy instruction. Schools are randomised to one of four groups: receiving either (i) the malaria intervention alone; (ii) the literacy intervention alone; (iii) both interventions combined; or (iv) control group where neither intervention is implemented. Children from classes 1 and 5 are randomly selected and followed up for 24 months. The primary outcomes are educational achievement and anaemia, the hypothesised mediating variables through which education is affected. Secondary outcomes include malaria parasitaemia, school attendance and school performance. A nested process evaluation, using semi-structured interviews, focus group discussion and a stakeholder analysis will investigate the community acceptability, feasibility and cost-effectiveness of the interventions.

**Discussion:**

Across Africa, governments are committed to improve health and education of school-aged children, but seek clear policy and technical guidance as to the optimal approach to address malaria and improved literacy. This evaluation will be one of the first to simultaneously evaluate the impact of health and education interventions in the improvement of educational achievement. Reflection is made on the practical issues encountered in conducting research in schools in Africa.

**Trial Registration:**

National Institutes of Health NCT00878007

## Introduction

There is increasing interest in strategies to improve education quality in poorly supported educational environments. Despite recent success in the expansion of educational access in low-income countries [1], concerns remain about levels of educational achievement and primary school completion in these countries, particularly those in sub-Saharan Africa. The reasons for this are multiple and complex, but it is increasingly recognized that poor health and nutrition affect children's cognitive functioning and therefore their ability to benefit from education [2]. Up to half of all school children in developing countries suffer from anaemia [3] and there is good evidence that anaemia affects children's cognitive abilities [4]. Many school children are also infected with parasitic worms and evidence suggests that those children who harbor heavy infections are found to perform poorly in tests of cognitive function [2]. Fortunately, iron supplementation and deworming have been showed to effectively improve cognitive performance and educational achievement [5-7]. These interventions can be cost-effectively delivered through school health and nutrition programmes which use the educational infrastructure to deliver interventions [8].

There is less quality evidence on how malaria may affect cognitive abilities and educational achievement or on how schools can tackle the problem of malaria among school children [9-11]. A randomised trial among Sri Lankan children showed that weekly malaria chemoprophylaxis with chloroquine improved school examination scores [12]. In a cluster randomised trial in Kenya, we previously evaluated the impact of intermittent preventive treatment (IPT) for malaria and found a 48% reduction in the rates of anaemia and a large effect size of 0.48 standard deviations (SD) on children's sustained attention in class [13]. Interestingly, no effect on educational achievement was observed. Possible explanations for such a finding are that children were not given the educational resources (such as textbooks or quality instruction) or a sufficient period of prolonged instruction to learn effectively during the time course of the evaluation. It remains highly plausible that the improved sustained attention observed in our study could translate into improved educational achievement, particularly in the early grades of school. Evidence suggests that executive function skills, such as regulation and attention, are particularly important for early achievement [14]. Thus, to achieve a measurable impact on education, it may also be necessary to improve teaching methods in order to capitalise on any improvements in health status of school children following malaria control.

Evidence on the effectiveness of interventions to improve educational quality has accumulated in recent years, with randomised trials showing that teacher incentives, student scholarships, providing textbooks, volunteer teacher aids and class size reduction can all boost student performance [15-22]. However, trials that assess the effectiveness of isolated interventions do not reflect the process of educational reform. Schools and education systems typically aim to improve quality by addressing several factors simultaneously, with the success of each one often dependent on the others. There is however very limited experimental evidence on how different interventions work together with one another to improve educational quality.

**Figure 1:**
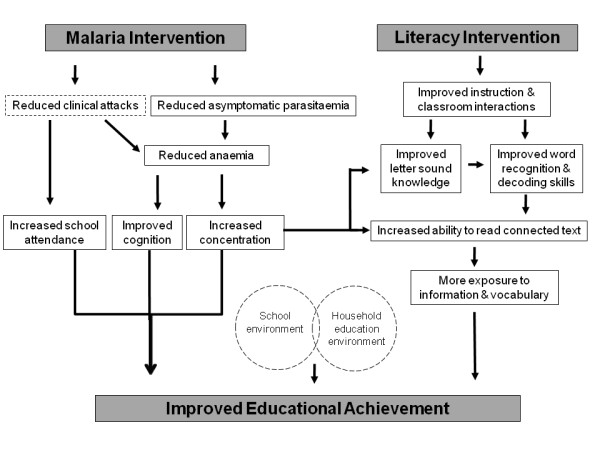
**The hypothesised causal pathways through which the malaria and literacy interventions are assumed to improve educational achievement**. Open rectangular boxes indicate secondary and mediating outcomes; the incidence of clinical attacks is not measured. Circle boxes indicate contextual variables measured at household and school levels.

In the current study, we aim to evaluate the impact of two interventions, one focused on malaria prevention and another on enhanced literacy instruction, on the educational achievement of school children in Kenya. Based on theories of how health interventions affect children's development and their education [2,23], we will assess the main outcomes along a hypothesised causal chain from malaria prevention to anemia, sustained attention and educational achievement (Figure 1), and thereby identify the channels through which the interventions are expected to operate [24]. Here, we discuss the rationale for the choice of interventions and present a novel factorial design of the study, investigating both health and educational interventions.

## Design of the interventions

Two interventions are being delivered through schools: (i) a malaria prevention strategy based on intermittent screening and treatment; and (ii) a literacy intervention based on a programme of training and support for class one teachers. Both interventions were developed within the context of current government strategies and guidelines, and were designed to be affordable and replicable on a large scale, within existing government programmes. In the control schools, no malaria or literacy interventions will be implemented, but these schools will receive the interventions at the end of the two year period.

### Malaria prevention

This intervention is based on intermittent screening and treatment (IST) for malaria. Every school term, all children will be tested for malaria using a rapid diagnostic test (RDTs). The RDT used is a ParaCheck-*Pf *device (Orchid Biomedical Systems, http://www.tulipgroup.com) which is able to detect *P. falciparum *and other (unspecified) *Plasmodium *species. Children (with or without malaria symptoms) found to be RDT-positive are treated with artemether lumefantrine (Coartem, Novartis), an artemisinin-based combination therapy. Testing and treatment is administered by district health workers and supported by the Division of Malaria Control, Ministry of Public Health and Sanitation. The first round of screening and treatment was conducted in March 2010, the second round in July 2010 and the third planned in September 2010. Further screening will also occur in 2011.

The intervention builds on our previous study evaluating the impact of school-based IPT [13]. In that study, all children received a full course of the anti-malarials sulfadoxine-pyrimethamine (SP) and amodiaquine (AQ) once a school term, irrespective of whether children are infected. However, changes in Kenya drug policy in 2009 led to the withdrawal of both AQ mono-therapy, because of future plans to combine the drug with artesunate for combination therapy, and SP, for which there are high levels of drug resistance in East Africa [25]. No other anti-malarials were identified as suitable for IPT in schools. Therefore, following extensive consultations with policy makers and malaria experts, the alternative of IST was identified. This intervention has recently been identified in the Kenya National Malaria Strategy, 2009-2017, under a newly launched *Malaria-free schools initiative *[26].

### Enhanced literacy instruction

The main components of the literacy intervention include: (i) a teacher manual, which includes 140 lessons for class one teachers develop literacy skills in English and Kiswahili; (ii) an initial three-day training workshop in term 1 and a follow-up one day workshop in term 2; and (iii) ongoing support which includes weekly interactive text messaging, and monthly written communiqués providing information and motivation. This intervention is based on a comprehensive survey of existing literacy instruction practices in the study area region (Dubeck et al. unpublished) and an analysis of how these practices can be developed to align more closely with current evidence on how best to promote successful literacy acquisition [27,28].

The lessons included in the teacher manual are designed to be used daily and are appropriate for developing beginning reading skills in an alphabetic language. They include letter-sound relationships, blending, spelling, connected text, developing a concept of word in text, phonological awareness, vocabulary, and reading comprehension. The 140 sequential lessons are structured to guide the teacher in what to say, what to do (i.e., with their hands or materials), which instructional materials to use, and the estimated time of the lesson. Understandably, there will be school days when the teacher will not instruct. The lesson plans were designed specifically for this study and based on extensive observation and interviews of existing teaching methods in the area over a 12 month period (Dubeck et al., unpublished). The plans build from existing teaching methods (e.g. choral repetition, use of song) and show teachers how these methods can be modified slightly to promote successful beginning reading instruction.

The initial training workshops were held in February and March 2010 and sought to provide class one teachers with background information about how children learn to read, to explain how to use the provided teacher manual and to give them the opportunity to customize materials for use in their classroom. Following the workshop, the study teams communicate weekly with teachers using text messages providing brief instructional tips and motivation to implement lesson plans. A response is required in order to receive a small amount of credit for their mobile phones which facilitates and provides an incentive for further communication. Each week teachers are requested to complete a Weekly Summary Sheet that documents which lessons they used, what worked well, and suggestions for improvement. A one-day, follow-up workshop was conducted in June 2010, when teachers learnt additional instructional methods, and received and shared feedback.

## Design of randomized evaluation

**Figure 2:**
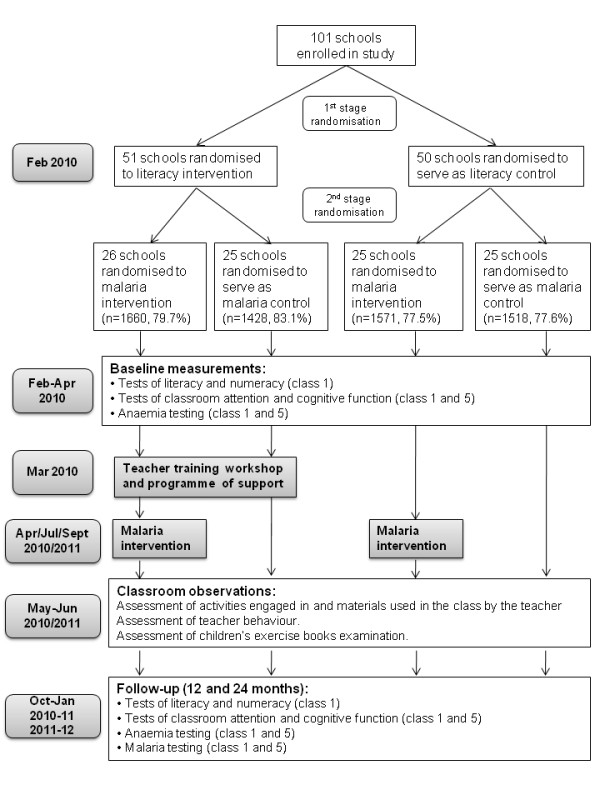
**Timetable and flowchart of randomisation and study design**. The number of children (and percentage of eligible children) randomised to each arm indicated.

The impact of the two interventions is being evaluated through a factorial design, cluster randomised trial, in which 101 schools are randomised to one of four groups: receiving either (i) the malaria intervention alone; (ii) the literacy intervention alone; (iii) both interventions combined; or (iv) control group where neither intervention will be implemented. Children from classes 1 and 5 are randomly selected and followed up for 24 months to assess the impact of the two interventions. Both classes receive the malaria intervention, but the literacy intervention is targeted only towards class 1 as this is when children learn to read. This is an unblinded study as, following randomization, schools are aware of whether or not they will receive the malaria or literacy interventions. The timeline and flowchart of the study design is shown in figure 2. A nested qualitative process evaluation is included to consider how the interventions work to improve educational achievement and to identify key assumptions and conditions underlying potential sustainability and scaling-up of the interventions. In this way, the evaluation not only addresses the question 'Does it work?', but also considers 'How does it work?', 'For whom?' and 'Under what circumstances?' [24,29,30].

The study's primary objective is to evaluate the impact of (i) school-based malaria prevention, using IST, and (ii) enhanced literacy instruction on the educational achievement of school children in Kenya. Secondary objectives are to (i) evaluate the impact of IST on anaemia and malaria parasitiaemia; (ii) evaluate the impact of enhanced literacy instruction in improving literacy rates of schoolchildren; (iii) to determine whether malaria and education interventions work synergistically together, such that learning is improved only when teaching is effective and children are healthy to benefit from it; and (iv) to investigate the cost-effectiveness and feasibility of the interventions.

### Study area and population

**Figure 3:**
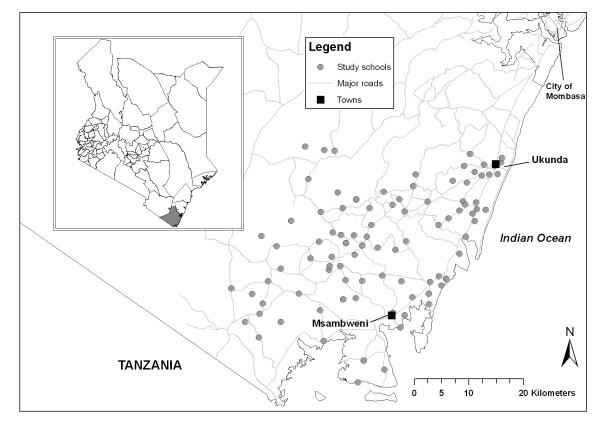
**Map of study areas in Kwale and Msambweni districts, Coastal Kenya**. Insert: Map of Kenya with Kwale and Msambweni districts shaded in grey.

The study is being conducted in rural government primary schools in Kwale and Msambweni districts, situated approximately 50 km south from Mombasa on the Kenyan coast (Figure 3). The study was carried out in these districts for several reasons. First, continuous precipitation supports intense year-round malaria (predominantly *Plasmodium falciparum*) transmission, with two seasonal peaks in malaria cases reflecting the bimodal rainfall pattern, with the heaviest rainfall typically occurring between April and June, with a smaller peak in October and November each year [31]. A 2008 survey among 20 schools in the study area found that up to 50% of school children harbor malaria parasites (Brooker, unpublished data), yet there are no initiatives specifically targeting malaria control in schools. Second, under-nutrition is common: the 2008 survey found that 21% of children were anaemic, reaching 38% in some schools (Brooker, unpublished data). Third, in terms of education, the area is one of the poorest in Kenya, having the worst mean national examination scores since 2005. The district is ranked as the seventh poorest district out of 76 districts in the country, and second poorest out of the seven districts in the Coast Province [32]. Around 80% of children attend primary school in these communities, but few proceed to secondary school.

There are 85 schools in Kwale District and 112 schools in Msambweni District. In Kwale District, a separate study is evaluating the impact of an alternative literacy intervention in two of the four zones; therefore only 20 schools in Mkongani and Shimba Hills zones were included in our study allowing the two interventions to proceed without leakage. In Msambweni District, we selected 81 of 112 schools; schools in Lunga Lunga and Mwereni zones about 70 km away from the project office, were excluded because of time and costs involve in travelling to them.

### Sensitization and recruitment

Sensitization took place at national, provincial and district levels before visiting the schools. At the national levels, the study was approved by the Division of Malaria Control, Ministry of Public Health and Sanitation and the Director of Basic Education, Ministry of Education. At provincial and district levels, meetings were held with the Provincial Medical Officer and the Provincial Director of Education in Mombasa, as well as district health and education officials in Kwale and Msambweni. Finally, school head teachers and Teachers' Advisory Centres (TAC) tutors were informed of the study.

Prior to randomisation, enumeration of children in all schools was carried out through school visits in January and February 2010. Subsequently, school meetings were held with parents and guardians of children to explain all aspects of the study, emphasizing that the participation of their children in the study was voluntary and they had the opportunity to opt out of the study at any time. There was an opportunity to ask questions. We then sought written consent from parents or guardians. If parents failed to attend these meeting, home visits were undertaken to obtain consent - see below. The eligibility criteria for inclusion into the study were as follows: enrolled at participating schools in classes 1 and 5; provision of informed consent from parent or guardian; and willingness of the child to participant. Exclusion criteria include parents or guardians unwilling to provide informed consent; an unwillingness of the child to participant; known allergy or history of adverse reaction to study medications; and known or suspected sickle-cell trait (these children were referred to testing and/or clinical management as per national guidelines).

### Randomisation

Randomisation was conducted in two stages, each involving public randomisation ceremonies. These ceremonies were considered important in assuring participating schools and stakeholders of the fairness and transparency of the allocation and represented a simple way of allocating schools to the four different groups (Figure 3).

In stage one, groups of schools were randomised either to receive the literacy intervention or to serve as a control school. In Kenya, schools are grouped by the District Education Office into so-called *school clusters *of between 3 and 6 schools, which regularly meet and share information, supported by a Teacher Advisory Centre (TAC) tutor. This randomisation was stratified by (i) cluster size, to ensure equal numbers of schools in the experimental groups; and (ii) average primary school leaving exam scores across the cluster, to balance the two groups for school achievement. District officials and representatives from all 26 school clusters were invited to a meeting, at which the objectives of the evaluation and randomisation procedures were described. Volunteers were chosen to represent the different school clusters and were asked to randomly draw envelopes each containing a cluster name from 10 pre-stratified ballot boxes and to sequentially place the envelopes in group A and group B. Volunteers then opened the envelopes to reveal which clusters were in groups A and B, but were not told which of group A or group B represented the literacy intervention group and which the control group. Subsequently, only intervention schools were informed of their participation in the literacy intervention, starting with the three-day training workshop. This randomisation procedure was designed to minimize contamination across clusters. It is still nonetheless possible that following the training workshop, teachers from the intervention schools will discuss their training with teachers from control schools. This is often unavoidable in studies evaluating education interventions and therefore, we will conduct classroom observations and interviews with teachers to assess the level of contamination in control schools in order to take this into account during analysis. However, it is unlikely that teachers from control schools will obtain the complete set of training materials.

In stage two, the malaria intervention was randomly allocated amongst the 51 schools allocated to the literacy intervention and the 50 schools allocated to serve as control schools during the first randomisation. Schools were stratified by average primary school leaving exam scores into 5 quintiles and by literacy intervention group, producing 10 strata overall. Prior the randomization ceremony, computer simulations were conducted to investigate the probability that all schools in a cluster could randomly receive the same malaria group allocation, thereby limiting the potential for independent analysis of the effects of literacy and malaria interventions. In this, 10,000 simulations were run using random numbers generated with Stata version 10 (Stata Corporation, College Station, Texas) to assign schools to malaria intervention and control groups. The results of these simulations showed that the probability of malaria allocation being the same across 8 or more of the 26 clusters was 0.1%, across 6 or more clusters was 2.1%, 4 or more was 17.2%, and 2 or more was 82.5%. On the basis of these simulations and because further restricting the randomisation process threatened the random nature of group allocation, it was decided to leave the coincidence of malaria and literacy group allocation to chance.

During the ceremony, the objectives of the evaluation and randomisation procedures were described to invited representatives from schools and local communities. Volunteers were then chosen to represent each of the 101 schools and asked to write school names on identical pieces of paper and include into identical envelopes. These were placed into ballot boxes based on the above system of restricted randomisation. The volunteers were asked to draw envelopes from each ballot box and sequentially allocate into two groups. Volunteers were not told which group was the intervention and which was the control, only that schools had been allocated to group C and group D. Contamination of the malaria intervention is unlikely since only children in the malaria intervention schools will be visited by district health workers and screened, and treated if found positive. Randomisation resulted in only one cluster where all schools received the same malaria group allocation.

### Intervention and follow-up

The school year in Kenya runs from January to end of November. The study commenced in January 2010 (Figure 3), following 12 months of piloting of the literacy intervention, educational assessments and questionnaires, and is scheduled to run two full school years. Baseline health and education surveys were conducted in intervention and control schools between January and March 2010, as described below. Teacher training workshops were held in February and March 2010 after baseline educational data was collected and the first round of malaria intervention delivered in March 2010. Any children who were absent on the day of the malaria intervention were revisited at school at a later date. A follow-up training workshop was held in June 2010, and the second and third rounds of malaria intervention conducted in July 2010 and October 2010. Class observations and checks on attendance were conducted between May to July 2010. The first follow-up survey will be carried out between October 2010 and January 2011, and the second follow-up survey 12 months later. During the second year of the study, only the malaria intervention will be delivered.

The school population is dynamic due to transfers, repetitions and drop-outs. We will not attempt to follow-up recruited children who transfer to schools outside the study area, but children who transfer to other study schools will be accounted for in the analysis. Children who repeat or drop-out will be included in the analysis up until the point they repeated or dropped-out, but excluded from the analysis thereafter.

## Outcomes

The primary outcomes are educational achievement and anaemia, the hypothesised mediating variables through which education is affected (Figure 1). These outcomes will be measured in a cohort of 6,000 children, comprising a random sample of 25 children in class 1 and 30 children in class 5 from each school, selected at baseline. A full range of educational outcomes are assessed in class 1 to evaluate the impact of both interventions, whereas a subset of educational outcomes is assessed in class 5 to evaluate the impact of only the malaria intervention.

In addition, we will assess secondary outcomes occurring along the hypothesized causal pathway (Figure 1), including malaria parasitaemia, school attendance and school performance, and will identify the channels through which the interventions are expected to operate. Intermediate variables, such as teacher knowledge, methods of instruction and classroom interactions, will be assessed during unannounced classroom observations. We will also assess important contextual factors, including school and household education environments.

Detailed process indicator data will be collected on the up-take and fidelity of programme implementation and, in the case of the literacy intervention, teacher attendance at professional development seminars, understanding of content, and implementation of lesson plans. Qualitative research on the acceptability, feasibility and cost-effectiveness of the two interventions will be evaluated by a semi-independent team of social scientists. This will seek to identify key assumptions and conditions underlying program sustainability and scaling-up, including organization and technical capacity of the government at national and local levels, based on focus group discussions and semi-structured interviews.

### Educational achievement and cognitive abilities

Children's competence in three main educational domains will be assessed at baseline, and 12 month and 24 month follow-up. Assessments are administered either as individual or group tasks.

Among children in class 1, literacy and numeracy tests are conducted in individualized and small-group settings. The literacy tasks focus on early literacy skills that are highly predictive of later reading acquisition [33], and include measures of oral vocabulary (receptive language), phonological awareness (matching beginning sound), letter knowledge, word recognition, passage reading, comprehension and spelling. The numeracy assessments measure foundational skills necessary for future understanding of mathematics, including numbers, operations, and geometry knowledge. In class 5, achievement tests were administered in groups of 15 or less and involved word recognition, sentence reading comprehension tests, and a written arithmetic test.

Among all children in both classes, sustained attention and non verbal reasoning are assessed. Among children in class 5, the sustained attention measure was the 'code transmission' adapted from the TEA-Ch (Tests of everyday attention for children) battery [34]. In the code transmission tasks, a list of digits is read out aloud at the speed of one every two seconds. Children are required to listen out for a 'code' - two consecutive occurrences of the number 5 - and then record the 2 numbers that preceded the code. Children are tested in groups of 15 or less, and given a warm-up exercise to familiarize them with the recorded voice and 3 practice exercises before each test. For children in class 1, floor effects were found to be common in the code transmission test. Instead, sustained attention was measured using the pencil tapping task in which children are required to tap a pencil on the desk a predetermined number of times in response to the assessor's taps. This task is conducted with predetermined delays between items and assesses both sustained attention and executive control. Finally, non verbal reasoning was assessed in class 1 by the Raven's Progressive Matrices task [35].

In total, 13 tasks are assessed in class 1: receptive language, spelling, beginning sounds, letter knowledge, word recognition, passage reading with comprehension, non-verbal reasoning, sustained attention, and five math tasks. Five tasks are assessed in class 5: word recognition, sentence reading comprehension, spelling, arithmetic and sustained attention. All of these tasks are included in the baseline and endpoint data collection. A limited number of these tasks will be used as a mid-point assessment.

All instruments were adapted to the Kenyan context to ensure face validity and appropriate stimuli over a period of 5 months (June-November, 2009). The provisional battery of tests was administered in 5 schools to provide pilot data to assess (i) properties of individual test items; (ii) internal reliability of individual tests; (iii) test-retest reliability of individual tests; and (iv) relationships between individual tests assessing related concepts. On the basis of these data, final changes were made to test items and a final battery of tests selected.

### Anaemia and malaria parasitaemia

Among all children, haemoglobin concentration is assessed at baseline and 12 and 24 months follow-up, based on a finger-prick blood sample using a portable photometer (Hemocue, Ängelholm, Sweden). Malaria parasitaemia will only be assessed at follow-up due to the ethical constraints of testing for malaria but not treating children found to be infected in the control schools. A finger-prick blood sample will be used to prepare thin and thick film for confirmation and quantification of malaria parasites on the basis of expert microscopy.

### School attendance

Attendance at school will be assessed through unannounced school visits made at three time points in term 2. Reasons for absence (e.g. illness, sent for fees, family emergencies, long-term absenteeism) will also be recorded.

### Teacher interviews and classroom observations

During the second school term, two unannounced visits to each school will be carried out to conduct teacher interviews and classroom observations. The teacher interview is based on a questionnaire developed in previous work in Western Kenya (Jukes MCH, Kim YS, Vagh SB: Class Size and Pedagogy: Which Teaching Methods are Crowded out by Free Primary Education in Kenya? in preparation) and on scenario-based questions adapted from the *Authentic Pedagogy *classroom observation tool [36].

The classroom observation involves an assessor observing class 1 English and Kiswahili lessons on two separate days and involves the integration of two approaches to classroom observation. Every 90 seconds a 'snapshot' of the classroom is taken, based in part on the Stallings snapshot instrument [37] and our adaptation of the instrument in previous work in western Kenya. The instrument codes the activities engaged in and materials used by the teacher and all students at one time point. In addition, specific literacy instruction practices are recorded at each time point based on established categories of effective pedagogy. This assessment is derived from the *CLASSIC *observation schedule designed to assess pedagogy for language instruction [38]. The instrument additionally includes teacher behaviours that are encouraged both during training and in the teachers' manual.

### Parental questionnaire

During consent, parents and guardian were asked to complete a parental questionnaire, which contained questions designed to assess the educational and socio-economic environment of children's households. Thirteen questions asked parents and guardians about the main languages they spoke in the household and to their children, their own reading ability and habits, their schooling, and involvement in their children's school. Nine questions asked parents about the ownership and use of mosquito nets by themselves and their children. Five questions asked parents about household construction and ownership of key assets, in order to provide proxy information on socio-economic status [39].

### School questionnaires

During school meetings, interviews with the head teacher collected information on the number of boys and girls enrolled in each class; examination results in English, mathematics and Kiswahili for the previous five years; indicators of the quality of infrastructure of the school, such as presence of toilets and hand washing facilities; whether the school had been in school health activities in the last year, such as school feeding, deworming and water and sanitation programmes; and the presence of health education material, including those for malaria. Of the 101 schools, all had recently received deworming and 48 have school feeding programmes, but none had implemented any malaria interventions.

The household and school level information will subsequently be used in the analysis to account for potential confounding, but also to explore the differential impact of the interventions.

## Process evaluation

A detailed process evaluation is being undertaken which aims to: (i) examine the implementation of the interventions; (ii) explore the context and fidelity of interventions; (iii) investigate community acceptability of interventions; and (iv) document factors external to the interventions which might impact upon both its implementation and its effectiveness. Such an evaluation will help the interpretation of results and help inform future large-scale implementation of the interventions.

First, a modified stakeholder analysis approach will be adopted to identify and assess the importance of key people, or groups of people who are likely to affect the implementation and longer-term sustainability of the programme [40,41]. Key stakeholders are likely to include: teachers, parents, children, community leaders, local health workers and education officers as well as individuals at provincial and central levels in the Ministries of Education and Health. Stakeholders from both intervention and control schools will be included in order to obtain views on the interventions, but also the acceptability of not immediately receiving the interventions. An assessment will be made of their importance to the success and sustainability of the programme and data on their views about the programme (e.g., expectations of the interventions, experiences of the intervention, acceptability of the approach, value to individuals & communities, impact on workload) will be collected through a series of focus group discussions as well as semi-structured interviews with people identified as key informants [42]. Discussions and interviews will be transcribed and translated, and content analysis using Nvivo 8 (QSR International, Melbourne, Australia) will be undertaken to identify themes based on people's experience and involvement in the intervention.

Second, an analysis will be undertaken of the structural, organisational and management factors that enhance or constrain effective implementation of the programme by staff from the Ministries of Health and Education. In addition to identifying and mapping these stakeholders through the stakeholder analysis, interviews will be conducted with purposefully selected key stakeholders in order to assess the organization and managerial capacities of the government at national and local levels [43]. These data will be analysed and interpreted iteratively based on implementation and organizational management theories [44] and the developed stakeholder analysis framework.

## Economic evaluation

An economic evaluation will be being undertaken alongside the trial, relating costs to a range of educational and health outcomes in the form of a cost and cost-effectiveness analysis. Costs will be assessed from both provider (government) and societal perspectives using an ingredient approach [45], based on interviews with individuals involved in delivering the interventions and by consultation of the programme accounting system. As the aim is to estimate the cost of scaling-up the interventions in Kenya, all costs associated with the evaluation will be excluded.

Cost-effectiveness analysis will consider improvements in test scores and educational achievement, assessed in terms of differences in standard deviation units. Changes in participation, attendance and educational achievement will be assessed in terms of percentage differences, and test scores in terms of differences in standard deviation units. CE will be calculated for each outcome and expressed as incremental cost-effectiveness ratios (ICER) in relation to the status quo, the other health/education intervention packages tested in the present study, and current interventions. Analysis will also incorporate improved cognition and learning into long-term outcomes, e.g. future earning streams [46], as well as examination of gains in specific sub-groups. A comparison with alternative education interventions, especially those conducted in Kenya [6,18,19,47], will also be undertaken. The impact on health will be assessed in terms of cases of anaemia averted, with comparison made with other school-based parasitic control programmes [48-50].

## Statistical considerations

### Sample size

It is considered that a reduction of at least 25% in anaemia is needed if the intervention is to be considered to have public health value. In our previous study of IPT in western Kenya, we found a 48% reduction in anaemia [13], whilst other school-based interventions have observed reductions in anaemia ranging from 5 to 60% [2]. To achieve 80% power of detecting a 25% reduction in prevalence of anaemia, assuming a baseline prevalence of 20%, a between school intra-class correlation (ICC) of 0.2 and 50 children sampled per school, requires a sample size of 27 schools in each malaria intervention arm [51]. This gives a total sample size of 54 schools and 2700 children.

Educational achievement and cognitive tests sample size calculations were conducted separately for outcomes measures used in class 1 and class 5 using optimal design software [52]. For each calculation we assume an overall sample size of 100 schools with 25 children per school (2500 children overall) and consider mean differences in test scores between the 50 intervention schools and 50 control schools independently for the malaria and literacy interventions. For achievement tests, this is sufficient to detect an effect size of 0.21 standard deviation (SD) with 80% power assuming an ICC of 0.2 (ICC varied from 0.1 to 0.2 with mathematics and literacy tests in class 2 in 210 schools in Western Kenya; ICC is expected to be lower in class 1) and a correlation between baseline covariates and the final outcome of 0.7. This sample size is sufficient to detect an effect size of 0.17 SD for tests of sustained attention, which have a lower intra-class correlation of 0.1 (ICC was 0.07 in our previous study [13]). The marginal utility of increasing sample size is relatively small. Adding 25 schools to the sample would reduce the detectable effect size from 0.21 to 0.19 SD for achievement tests and 0.17 to 0.15 for attention tests.

An additional concern when calculating the sample size arose from the relatively low prevalence of malaria, estimated from recent school surveys to be around 15% (Brooker et al. unpublished). When a small sample (N = 25) are sampled from a large class, the possibility arises either that the proportion of children with malaria in the sample would be low relative to the overall sample and, for both large and small classes, that the absolute number of children with malaria in the sample would be small. To investigate this issue, we ran 10,000 simulations of such a random sampling exercise, assuming a uniform malaria prevalence of 15%, to determine the sample size required to ensure both of two eventualities with 80% certainty: a) the proportion of children with malaria in the sample is at least 75% of the proportion in the population and b) at least 3 children with malaria would be included in the sample in each class. Simulations suggested that a sample of 25 children would be needed for class up to 50 children in size, and a sample of 30 would be needed for large classes. This sample size was able to be achieved for class 5, but logistical constraints in conducting the battery of educational assessments meant that only 25 children could be sampled in class 1.

### Data analysis

The primary analysis will follow the intention to treat principle, whereby all children regardless of whether they received the full intervention or not will be included in the analysis. Data will be analysed both at the cluster level, by deriving summarizing summary statistics, and at the individual level (see [51]). The effect of each intervention will, in the first instance, be analysed separately.

Weighted estimates will be provided, with weights proportional to the number of schools per stratum. For binary outcomes, the overall risk for each school will be presented by intervention group and linear regression used to estimate unadjusted relative risk and 95% confidence intervals (CI) associated with each intervention. For continuous outcomes, means for each school will be shown and the arithmetic mean and SD of mean values and scores and associated 95% CI for each intervention group calculated. Again, linear regression will be used to estimate mean difference and 95% CI for each intervention.

In addition to estimation of crude intervention effects, adjusted analysis will be performed to account for potential confounding variables measured at baseline. Depending on the outcome, logistic or linear regression will be conducted at the individual level, ignoring the clustering of the data and intervention effect. In each case, the outcome will be regressed on baseline predictors and the resultant residuals will then be used as the summary measure for each school based on a comparison of the observed outcome in that school and predicted outcome in the absence of an intervention effect. Systematic differences in the residuals by intervention groups will provide a measure of intervention effect.

As well as cluster-level analysis, individual-level analysis using suitable generalised linear models will be undertaken to adjust for clustering by school [51]. Random-effect models will also be used to explore whether there are any differences in impact of the interventions according to child age, sex, home environment, school quality as well as differences in the uptake of each intervention.

Once the separate effects of each intervention have been analysed, the joint effects of the two interventions will be examined at the cluster level. In its simplest form, these will be evaluated by incorporating interaction terms into the regression model. If a significant interaction is present, then each intervention effect will be reported separately in the presence and absence of the other intervention. If sample size permits, individual-based regression analysis of interaction effects will be examined.

## Ethical considerations

### Ethical approval

The study was approved by the Kenya Medical Research Institute and National Ethics Review Committee (SSC No. 1543), the London School of Hygiene and Tropical Medicine (LSHTM) Ethics Committee (5503), and the Harvard University Committee on the Use of Human Subjects in Research (F17578-101). Sponsorship and insurance is provided by the LSHTM's Clinical Trials Sub-Committee (QA225).

### Information and informed consent

Written and verbal information is provided in English and Kiswahili. Documents translated into Kiswahili were checked through back-translation. Prior to the onset of the study, school meetings were held with the parents/guardians of children enrolled in participating classes to describe the purpose of the study, the procedures to be followed, the risks and benefits of participation. Information sheets are provided to the parents/guardians for their review. Individual informed consent is obtained from parents/guardians of selected children, and is recorded by signature or thumb-print. For those parents who not attend these meetings, follow-up was made through community leaders and household visits. Parents/guardians had the chance to ask questions and were asked to provide signed informed consent for their child to participate in the study. They were also informed that participation of their child in the study is completely voluntary and that they may withdraw from the study at any time. At each data collection, verbal assent is sought from children.

### Trial oversight

Ethical and safety aspects of the study are overseen by an independent data safety and monitoring board (DSMB) consisting of experts in clinical medicine, epidemiology, and statistics, who met convened to the start of data collection and will convene at the completion of the study to approve the final data analytical plan. A local clinician based at the KEMRI-Wellcome Trust Research Programme in Kilifi, two hours from the study area, acts as a Local Safety Monitor monitors and reports to the DSMB chair any severe adverse effects arising from the malaria treatment.

Community and school mechanisms have been established to facilitate a two-way flow of information between the study team and the schools and communities. These mechanisms include feedback letters to schools, school meetings, community meetings and meetings with local chiefs and leaders, as well as regular reporting to health and education officials at district, provincial and national levels. This system of communication, especially community meeting, is particularly important in control schools in order to prevent attrition or any non-cooperation for outcome assessment.

## Discussion

It is well accepted that decisions regarding health, education and social interventions should be based on robust evidence on the benefits of the proposed intervention. Cluster randomised trials, whereby groups of individuals may be randomly allocated to different interventions or a counterfactual (typically a control group), have the potential to provide unbiased estimates of the impact of interventions which are delivered at the community level [51]. More recently, there has been an increasingly appreciation that studies should not evaluate what interventions work, but also why it works, and so enhancing the policy relevant of evaluations [24,30,53]. The present evaluation adopts a theory-based approach to investigate the causal chain through which the intervention is expected to have its intended impact (Figure 1). We also employ both quantitative and qualitative methods to understand the context and process of the interventions, but also to understand who benefits most from the interventions and who benefits least [30].

Conducting research in schools, especially in an African context, raises a number of practical issues. First, in order to maximize the policy relevance it is essential to understand the country context of the research, the related policymaking processes and to engage key stakeholders [54]. We sought to actively engage with the education and health authorities at national, provincial and district levels. Our study arose from a desire from the Government of Kenya to have better evidence on the benefits of school-based malaria control and the interactions of malaria control with interventions to improve education quality. Specifically, the Ministry of Education, recognising malaria as a leading health problem facing school children and affecting education, sought clear policy recommendations as to the optimal approach to incorporate malaria control into its school health package. Meanwhile, the health sector recognised the importance of malaria control for school-aged children, and in 2009, the Ministry of Public Health and Sanitation launched its new National Malaria Strategy, 2009-2017, a new key component of which was a *Malaria-free Schools Initiative *[26]. Therefore, we held a series of meetings with government officials to discuss possible study designs. Once the design was finalized, we held regular meetings with the district educational and health officials to gain their support and to organize the logistics of school selection and data collection. The involvement of local officials was particularly important in the public randomisation ceremonies.

Second, it is essential to conduct adequate sensitization with local communities and community leaders, and not just with schools and parents. This is because opinion leaders in the community can influence involvement in the study. Gaining community trust is a lengthy process, but essential to the study's success. Third, it is important to obtain a reliable census of children enrolled in school. School populations are very dynamic, with children transferring between schools, dropping out and repeating classes. Because of this fluidity, school registers do not always provide up-to-date information. We therefore undertook a school census prior to consenting, but also found that this needed to be subsequently updated since some children repeated years half way through the school term. Fourth, during data collection, it was important to coordinate activities so as to minimize disruption to teaching and school activities.

Fifth, school-level cluster randomized trials require large sample sizes. This is especially the case with educational outcomes which vary from one school to another. It is essential to measure the intra-class correlation coefficient of such outcomes in order to derive precise estimates of required sample size. We found that, although 100 schools may be insufficient to detect very small effect sizes (< 0.2 SD), the marginal utility of increasing the number of schools further was small. Efforts to improve precision of outcome measures could alternatively be invested in expanding the assessment of baseline measures likely to predict final outcomes [55].

Finally, contamination between schools was a concern and transfers of children between schools may also dilute the effect of the interventions. This was a particular concern for the education intervention. To reduce the potential for contamination, we randomised the education intervention by school cluster and are also measuring possible contamination through unannounced classroom observations.

Despite these issues, the study is well underway and the interventions appear to be practical feasible and popular with the local communities. A particular strength of our study is the strong policy linkages with both health and education sectors in Kenya. These sectors are seeking clear policy and technical guidance as the optimal approach to malaria control in schools as well as evidence that systematic instruction is essential for progress in early grading reading and educational achievement overall. The current evaluation, the first of its kind in Africa, will address this policy information gap. In addition, the work has the potential to raise awareness of the need to improve both health and instruction quality to enhance education in Kenya, and also other countries where malaria is common and literacy poor.

## Competing interests

The authors declare that they have no competing interests.

## Authors' contributions

SB secured the funding and is responsible for the overall study design and project management, and drafted the manuscript. GO is responsible for coordination and supervision of fieldwork. KN participated in the study design and coordination. MMD designed the education intervention and coordinated the development and implementation of educational assessments. KEH is responsible for coordination of data management and fieldwork. HI contributed to the design of the education intervention and assessments. MCHJ is responsible for the overall study design and the design and implementation of the educational assessments. All authors read and approved the final manuscript.
